# The Dependence between Urinary Levels of Angiogenesis Factors, 8-Iso-prostaglandin F2*α*, *ɣ*-Synuclein, and Interleukin-13 in Patients with Bladder Cancer: A Pilot Study

**DOI:** 10.1155/2020/4848752

**Published:** 2020-12-02

**Authors:** Beata Szymańska, Ewa Sawicka, Michał Matuszewski, Janusz Dembowski, Agnieszka Piwowar

**Affiliations:** ^1^Department of Toxicology, Faculty of Pharmacy, Wroclaw Medical University, Wroclaw, Poland; ^2^Department of Urology and Oncological Urology, Faculty of Medicine, Wroclaw Medical University, Wroclaw, Poland

## Abstract

During the last decade, a significant increase in the incidence of bladder cancer (BC) has been observed. Angiogenesis plays a key role in the process of tumor growth and metastasis. Additionally, the participation of oxidative stress and chronic inflammation in BC pathogenesis is indicated. The aim of the study was to evaluate the urinary levels of parameters of angiogenesis, stimulating angiogenin (ANG) and inhibiting angiostatin (ANGST), 8-iso-prostaglandin F2*α* (8-iso-PGF2*α*) as a marker of oxidative stress, *ɣ*-synuclein (SNCG) as a cancer progression parameter, and interleukin-13 (IL-13) as an anti-inflammatory immunomodulator. The levels of ANG, ANGST, 8-iso-PGF2*α*, SNCG, and IL-13 in the urine of BC patients and healthy controls were measured by the enzyme-linked immunosorbent assay. These parameters were examined in the whole group of BC patients and in subgroups depending on the clinical stage: nonmuscle-invasive bladder cancer (NMIBC) and muscle-invasive bladder cancer (MIBC); histopathologic malignancy: low grade (LG) and high grade (HG) and in primary and recurrent BC. Significantly, higher urinary parameters were found **i**n BC patients in comparison to controls. Levels of all parameters increased with the development of cancer, with the exception of 8-iso-prostaglandin F2*α*, in which the level was higher in the early stages of the disease, but these differences were not statistically significant. Some correlations have been demonstrated between parameters in BC patients. Based on the receiver operating characteristic curves, ANG and ANGST had the best diagnostic value for BC. The obtained results indicate the important role of the examined parameters of angiogenesis, oxidative stress, and inflammation in the pathogenesis and development of BC. It is reasonable to continue research in order to thoroughly assess the impact of various associated processes on the course of BC. It is also important to carry out similar tests in patients with other urological diseases.

## 1. Introduction

Bladder cancer (BC) is the 10th most common type of cancer worldwide, with an estimated 549,000 new cases and 200,000 deaths in 2018. BC is more common in men than in women, with respective incidence and mortality rates of 9.6 and 3.2 per 100,000 in men (about 4 times higher than for women around the world). Thus, the disease ranks higher among men, in whom it is the sixth most common cancer and ninth leading cause of cancer death. Incidence rates in both sexes are highest in Southern Europe (Greece, with the highest incidence rate in men globally; Spain; Italy), Western Europe (Belgium and the Netherlands), and Northern America [1]. The main reasons that may explain the higher incidence of BC in more developed countries are faster economic development, intensification of BC risk factors, including smoking, poor eating habits, and the detrimental effects of environmental pollution, or occupational exposure [2].

Diagnosis of BC is primarily based on invasive cystoscopy and urinary sediment cytology. Although urinary cytology is used for screening for BC, it is sometimes difficult to judge cytologic specimens, particularly for low-grade cancers, while cystoscopy is invasive and burdensome for patients and expensive for healthcare providers [3]. Therefore, new, noninvasive methods for BC detection would open up new possibilities for the diagnosis and monitoring of this disease. Despite the availability of many laboratory biochemical parameters, no marker that would significantly improve the diagnosis of this disease has been found. Therefore, all studies in this area seem to be justified [4].

The development and metastasis of tumors are especially associated with the process of angiogenesis. Tumor angiogenesis occurs when there is an imbalance between factors stimulating and inhibiting this process in favor of proangiogenic factors [5]. Some literature data indicate that tumor angiogenesis, i.e., the formation of new blood vessels within the tumor, plays a significant role in BC pathogenesis [6].

Angiogenin (ANG) is a potent stimulator of new blood vessels through the process of angiogenesis. The study noted the involvement of ANG in the tumor formation process. In addition, there is evidence to support the synthesis of ANG in cancer cells [7]. High levels of this proangiogenic parameter have been found in various cancers: colorectal, breast, kidney, and malignant melanoma. This fact and the demonstration of the relationship between angiogenin and the degree of tumor aggressiveness may indicate a relationship between ANG and the development of cancer, including BC [8].

Angiostatin (ANGST), the N-terminal fragment of plasminogen, is a powerful antiangiogenic factor produced by the cleavage of plasminogen by several enzymes such as urokinase and tissue plasminogen activator. The role of angiostatin is to inhibit the proliferation and induction of apoptosis of vascular endothelial cells, which can lead to the inhibition of tumor growth. It can also be used in radiotherapy for cancer because of its strong ability to inhibit tumor growth and create new vessels. The function of angiostatin is also to inhibit the activation and migration of neutrophils, which indicates its anti-inflammatory properties [9].

The impact of oxidative stress (OS) on BC development is associated, among other things, with the maintenance of an inflammatory microenvironment that promotes increased proliferation of cancer cells. Studies show that patients with BC have increased lipid peroxidation, and the products resulting from this process affect not only BC initiation (through DNA damage as well as lipid and protein oxidation) but also the further stages of cancer progression [10, 11]. Biomarkers reflecting the process of lipid peroxidation are the most-examined F2-isoprostane and 8-iso-prostaglandin F2*α* (8-iso-PGF2*α*). Belonging to this group of compounds is considered a reliable parameter for assessing lipid peroxidation in many diseases, such as neurodegenerative diseases, multiple sclerosis, hypercholesterolemia, diabetes, autoimmune diseases, and cancers [12].

Studies show that protein-*ɣ*-synuclein (SNCG) overexpression is an important factor in cancer pathogenesis, stimulating tumor progression through a variety of mechanisms such as promoting cell proliferation and chromosome instability, cell invasion, and metastasis [13]. SNCG is expressed in various types of cancers, and measurement of its levels can be useful as a prognostic parameter in the early identification of the tumor process. This is a factor that can predict unfavorable prognosis in breast, colorectal, and pancreatic cancer, esophageal cancer, prostate, and stomach cancer [14]. Studies on the role of this parameter in bladder cancers have been started [15, 16].

Interleukin-13 (IL-13) is a pleiotropic cytokine with anti-inflammatory and immunoregulatory activity [17]. Literature data indicate its role in the pathogenesis of cancer, such as breast, ovarian, pancreatic, colorectal, head, and neck cancers and lymphoma cancers [18, 19]. However, the results of some studies indicate a contradictory role for IL-13 in promoting and fighting the progression of cancer. The involvement of IL-13 in the escape of tumor cells from host immune surveillance is important. The use of IL-13 inhibitors in targeted immunotherapy in cancers is also being considered [20].

The aim of the study was to determine the level of ANG and ANGST as parameters of angiogenesis, 8-iso-PGF2*α* as an indicator of oxidative stress, SNCG as a stimulator of tumor progression, and IL-13 as an inflammatory immunomodulator in the urine of patients with bladder cancer and to estimate their potential as possible diagnostic parameters in these patients.

## 2. Materials and Methods

### 2.1. Patient Selection and Collection of Data

The study group consisted of BC patients in the Urology and Oncological Urology Department (Wroclaw Medical University) during the period from 2014 to 2015. The control group was selected from participants with no history of cancer or other chronic inflammation, which was excluded by clinical examination of the cytology of urine sediment and a urine strip test. All participants were informed of the aim of the study and gave written consent to participate. The study was approved by the Ethics Committee of Wroclaw Medical University (KB-292/2-16).

Based on histopathological examination of tissues (performed in the Department of Pathomorphology and Oncological Cytology, Wroclaw Medical University), patients were divided into subgroups according to tumor grade: low grade (LG) and high grade (HG)—on the basis of the WHO/International Society of Urological Pathology—ISUP System 2004, into subgroups of NMIBC (nonmuscle-invasive bladder cancer) and MIBC (muscle-invasive bladder cancer), according to the TNM classification (tumor, noduli, and metastases) developed by the Union for International Cancer Control (UICC) in 2009, and additionally according to whether BC was primary or recurrent [4].

### 2.2. Materials and Methods

The material for the laboratory test examination of selected parameters consisted of urine from BC patients and the control group. The urine samples were collected in polystyrene containers (Aptaca, Italy) and then centrifuged by an MPW-350 laboratory centrifuge (MPW Instruments, Poland) for 10 minutes (1500 xg), after which the obtained supernatant was removed, placed in Eppendorf tubes, and stored at −80°C for further investigation.

Concentrations of selected/chosen parameters were measured in urine by the immunoenzymatic method (ELISA) with enzyme-linked immunosorbent assay kits: Human Angiogenin ELSA Kit, Cusabio, CRL, Human Angiostatin ELISA Kit, Genorise Scientific, USA, 8-Iso-PGF2*α*—OxiSelect 8-iso-Prostaglandin F2*α* ELISA Kit, Cell Biolabs, USA, Human Gamma-Synuclein (SNCG) ELISA Kit, Cusabio, CRL, and Human IL-13 Platinum ELISA IL-13, eBioscience, Austria, according to the manufacturer's instructions in a listed test.

Concentrations of parameters were calculated in relation to the urine creatinine concentration estimated by Jaffe's routine method based on the reaction of picric acid (Picric Acid, USA, Sigma, Cat. no. 319287).

### 2.3. Statistical Analysis

Statistical analysis was conducted with Statistica PL software (version 13.3). The normality of distribution was checked by Lilliefors and Kolmogorov–Smirnov tests. The nonparametric *U* Mann–Whitney and Kruskal–Wallis tests were used for the comparison of the variables between groups, and the post hoc analysis (Bonferroni test) was used for nonparametric data. The associations between continuous variables were analyzed by the Spearman test. The receiver operating characteristic curves (ROCs) were estimated. The area under the curve (AUC) and best cutoff point were calculated. Diagnostic value indicators with 95% CI such as sensitivity, specificity, positive predictive value (PPV), negative predictive value (NPV), and likelihood ratio (LR) were calculated. The values of *p* < 0.05 were considered as statistically significant.

## 3. Results

### 3.1. Study Population

The study group consisted of 60 BC patients including 50 men and 10 women with a mean age of 66 years. The control group included 28 healthy volunteers, 23 men and 5 women with a mean age 67 years. No statistically significant differences in characteristic features were observed between patients and the control group. The demographic and clinical characteristics of the examined groups are shown in Table 1. Age and sex of BC patients and the control group were not different (*p* > 0.05).]

### 3.2. Comparison of the Level of Urinary Parameters in BC Patients with the Control Group

**Figure 1:**
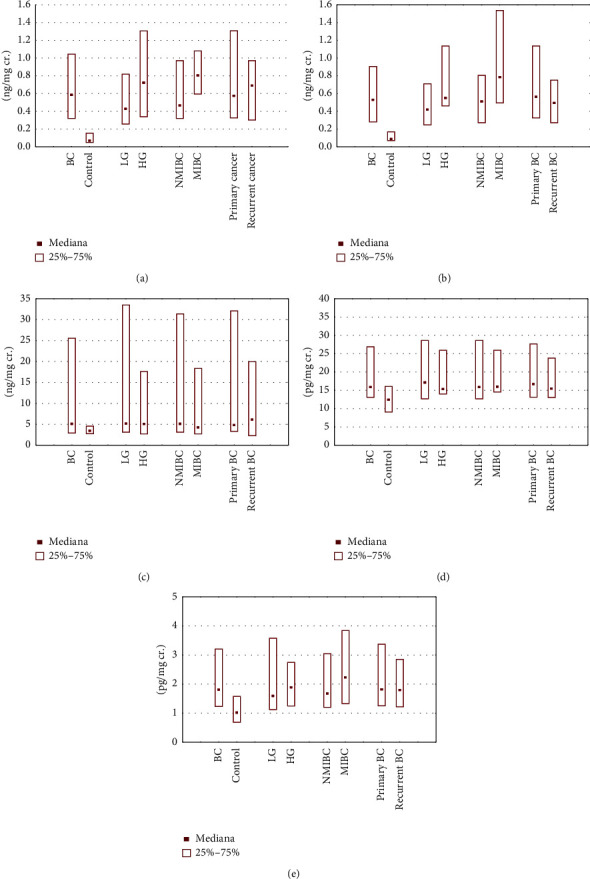
Median levels and interquartile ranges of examined parameters in the urine in all patient groups and the control group: (a) angiogenin, (b) angiostatin, (c) 8-iso-prostaglandin F2*α*, (d) *ɣ*-synuclein, and (e) interleukin-13.

Median levels and interquartile ranges in BC patients and the control group, with statistical analysis of examined parameters: ANG, ANGST, 8-iso-PGF2*α*, SNCG, and IL-13 in the urine, are presented in Table 2 and Figure 1.

As shown in Table 2, median levels of ANG, ANGST, 8-iso-PGF2*α*, SNCG, and IL-13 were 9.3-fold, 6.6-fold, 1.5-fold, 1.3-fold, and 1.8-fold higher, respectively, in the BC patients compared with the control group. There were statistically significant differences between the levels of all examined parameters (Table 2).

### 3.3. Assessment of the Diagnostic Value of Urinary Parameters

**Figure 2:**
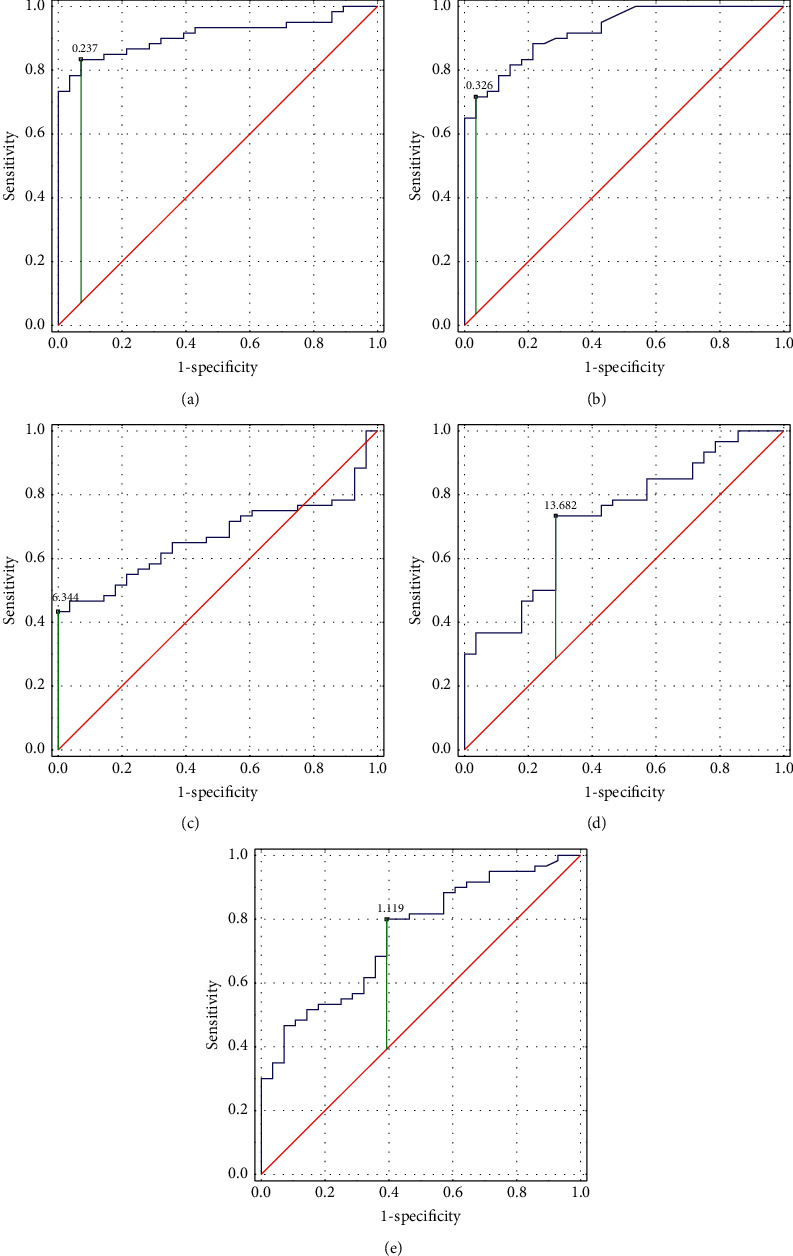
ROC curve analysis for examined parameters: (a) angiogenin, (b) angiostatin, (c) 8-iso-prostaglandin F2*α*, (d) *ɣ*-synuclein, and (e) interleukin-13.

The ROC curve analysis of ANG, ANGST, 8-iso-PGF2*α*, SNCG, and IL-13 conducted for examined parameters showed an area under the curve (AUC) of 91% (95% confidence interval (CI) (85–97%), *p* < 0.001)), 92% (95% CI (87–98%), *p* < 0.001)), 67% (95% CI (56–78%), *p*=0.003)), 73% (95% CI (62–84%), *p* < 0.001)), and 75% (95% CI (65–85%), *p* < 0.001)), respectively (Figure 2).

Sensitivity, specificity, PPV, NPV, LR+, and LR−for each of the examined parameters are shown in Table 3.

The cutoff value for ANG, ANGST, 8-iso-PGF2*α*, SNCG, and IL-13 was 0.24 ng/mg creatinine, 0.33 ng/mg creatinine, 6.34 ng/mg creatinine, 13.68 pg/mg creatinine, and 1.12 pg/mg creatinine, respectively.

### 3.4. Relationships between Parameters

In BC patients, significant positive correlations between parameters, ANG vs. ANGST (*R* = 0.67, *p* < 0.001), SNCG vs. IL-13 (*R* = 0.60, *p* < 0.001), 8-iso-PGF2*α* vs. IL-13 (*R* = 0.47, *p* < 0.001), ANGST vs. 8-iso-PGF2*α* (*R* = 0.41, *p*=0.001), ANGST vs. IL-13 (*R* = 0.40, *p*=0.002), and ANGST vs. SNCG (*R* = 0.36, *p*=0.004), were found.

### 3.5. Dependence of the Parameter Level on the Degree of Malignancy and Invasiveness of BC

Analysis of the levels of the examined parameters was conducted within subgroups of BC patients divided according to the clinical stage of malignancy. Median levels and interquartile ranges of parameters in the urine of BC patients in the LG and the HG subgroups and the control group, with statistical analysis, are presented in Table 4 and Figure 1.

Median levels of ANG, ANGST, 8-iso-PGF2*α*, SNCG, and IL-13 were 7-fold, 5-fold, 1.5-fold, 1.4-fold, and 1.6-fold higher, respectively, in the LG subgroup of BC patients compared with the control group. There were statistically significant differences between the levels of all parameters in the LG subgroup of BC patients and those of the control group. Median levels of ANG, ANGST, 8-iso-PGF2*α*, SNCG, and IL-13 were 12-fold, 6.6-fold, 1.5-fold, 1.2-fold, and 1.9-fold higher, respectively, in the HG subgroup of BC patients compared with the control group. There were statistically significant differences between the levels of parameters in the HG subgroup of BC patients and in the control group without 8-iso-PGF2*α*. However, statistical analysis showed no significant differences between any parameter levels in the LG or the HG subgroups of BC patients (Table 4).

Median levels and interquartile ranges of parameters in the urine of BC patients in the NMIBC and the MIBC subgroups and the control group, with statistical analysis, are shown in Table 5 and Figure 1.

Median levels of ANG, ANGST, 8-iso-PGF2*α*, SNCG, and IL-13 were 7.7-fold, 6.3-fold, 1.5-fold, 1.3-fold, and 1.7-fold higher, respectively, in the NMIBC subgroup of BC patients compared with the control group. There were statistically significant differences between the levels of all parameters in the NMIBC subgroup of BC patients and those in the control group. Median levels of ANG, ANGST, 8-iso-PGF2*α*, SNCG, and IL-13 were 13.3-fold, 9.8-fold, 1.2-fold, 1.3-fold, and 2.2-fold higher, respectively, in the MIBC subgroup of BC patients compared with the control group. There were statistically significant differences between the levels of examined parameters in the MIBC subgroup of BC patients and the control group, except for 8-iso-PGF2*α*. The analysis showed no significant differences between any parameter levels in the NMIBC or the MIBC subgroups of BC patients (Table 5).

### 3.6. Differences in the Level of Parameters in Primary and Recurrent BC

Median levels and interquartile ranges of parameters in the urine of BC patients in the subgroup of primary BC, the subgroup of recurrent BC, and the control group, with statistical analysis, are presented in Table 6 and Figure 1.

Median levels of ANG, ANGST, 8-iso-PGF2*α*, SNCG, and IL-13 were 9.5-fold, 7-fold, 1.4-fold, 1.3-fold, and 1.8-fold higher, respectively, in the subgroup of primary BC patients compared with the control group. There were statistically significant differences between the levels of parameters in the subgroup of primary BC patients and the control group without 8-iso-PGF2*α*. Median levels of ANG, ANGST, 8-iso-PGF2*α*, SNCG, and IL-13 were 11.3-fold, 6.1-fold, 1.7-fold, 1.2-fold, and 1.8-fold higher, respectively, in the subgroup of recurrent BC patients compared with the control group. There were statistically significant differences between the levels of parameters in the subgroup of primary BC patients and those of the control group, except for 8-iso-PGF2*α*. Additionally, the analysis showed no significant differences between any parameter levels in the NMIBC and the MIBC subgroups of BC patients.

## 4. Discussion

Angiogenesis is an essential part of many physiological processes, but it is also critical to tumor growth and tumor cell survival as it ensures a constant supply of oxygen and nutrients. In the case of cancer, this condition is disturbed, and there is a shift towards proangiogenic factors [6].

In our research, the results were quite surprising because the levels of ANG and ANGST in the urine of BC patients were similar, which may indicate a balance between these pro- and antiangiogenic parameters. However, it should be noted that these parameters were determined in the urine of patients and reflected the local urothelial BC angiogenesis process. An increase in the levels of ANG and ANGST was observed with an increase in the invasiveness and malignancy of cancer (MIBC and HG), but it was not statistically significant. In primary cancer and recurrence, ANG and ANGST levels were similar. ROC analysis also indicated ANG and ANGST to be the parameters with the best diagnostic value in BC among those examined. The studies showed a strong mutual positive correlation between ANG and ANGST (*R* = 0.67, *p* < 0.001). This confirms the significant role of angiogenesis processes in the development and metastasis of tumors [6].

Peres et al. [21], in studies on human BC cell lines (UROtsa, RT4, RT112, 5637, UM-UC-3, T24, TCCSUP, and UM-UC-14), proved that high ANG expression correlated with the increased matrix activity of metalloproteinase-2 (MMP2). In patients with MIBC, they showed higher levels of ANG and MMP2 and lower levels of these parameters compared to NMIBC. The average relative levels of mRNA for both ANG and MMP2 in invasive BC were significantly increased compared to the average relative levels of ANG and MMP2 mRNA in noninvasive BC.

Urquidi et al. [22] examined the concentration of ANG in the urine of a group of patients with active BC and people with benign urological disorders. They observed a significantly higher level of ANG in BC compared to benign urological diseases. Angiogenesis plays a role in both local tumor development and distant metastases. ANG appears to be a key stimulant in the process of angiogenesis, enabling tumor growth as well as metastasis.

No information was found in the available literature regarding the research into ANGST in BC, so our observations are innovative.

In our research, the effect of oxidative stress on the cancer process in the bladder was observed, and the elevated level of 8-iso-PGF2*α* in the urine of BC patients suggests an increase in lipid peroxidation, especially in the early stages of the disease. Based on the analysis of our own results carried out in subgroups of BC patients with different degrees of clinical advancement and histopathological malignancy, higher average 8-iso-PGF2*α* values were found in benign BC (NMIBC and LG) conditions compared to advanced ones (MIBC and HG), but they were not statistically significant. Additionally, the 8-Iso-PGF2*α* values in recurrent BC were slightly higher than in primary BC, although not statistically significant.

Verratti et al. [23], using the immunocytochemical method, examined the expression of 8-iso-PGF2*α* in the healthy urinary bladder tissue and the tissue derived from BC. Release of 8-iso-PGF2*α* was significantly reduced in the tumor tissue compared to the healthy bladder tissue. Inhibiting the production of a strong vasoconstrictor such as 8-iso-PGF2*α* in the vascular homeostatic mechanism of the bladder may reflect tumor response, tending to contrast the vasoconstrictor effect and the need to support oxygen supply to cancer cells.

SNCG is secreted from different tumor cells. It is a parameter that has recently aroused interest among researchers. Only single literature study indicates the usefulness of this marker in the diagnosis of bladder cancer [16].

Mhawech-Fauceglia et al. [24], using enzyme immunoassay and western blotting, were the first to discover high SNCG expression in NMIBC and confirm its high diagnostic performance by high AUC value. The researchers also showed, for the first time, a relationship between high SNGC expression and the tumor stage in BC patients. Additionally, the authors indicated that urine SNCG can discriminate BC from other urinary diseases and is a useful prognosticator of postsurgical recurrence.

Liu et al. [16] revealed that the level of SNCG in urine among patients who had BC tumor resection was significantly reduced. In addition, in patients who were to experience a relapse in the future, the marker concentration was higher than among those who did not relapse.

Zhao and Xing [15] studied the SNCG expression, using immunohistochemistry, in tissues from BC patients and revealed statistically significantly higher expression of this parameter in samples taken from patients than in the tissues of healthy individuals, especially at higher stages of cancer.

In our study, SNCG levels were statistically significantly higher in BC and in all analyzed subgroups compared to controls; however, no significant differences were found between the initial and more advanced stages of BC. The studies showed a strong mutual positive correlation between SNCG and IL-13 (*R* = 0.60, *p* < 0.001).

Our research has shown higher levels of IL-13 in more advanced stages of the disease (HG and MIBC). The increased presence of IL-13 in the urine of BC patients was probably due to the increased inflammatory process associated with cancer, which includes local changes in the urinary bladder or may have its origin in the tumor itself. Tumor cells produce IL-13 in response to the host's defense response and defend against apoptosis [25].

Margel et al. [26] showed an elevated concentration of IL-13 in the urine of BC patients compared to the control, but no statistically significant differences between MIBC and NMIBC were revealed, although higher IL-13 values were obtained for MIBC, similarly to our studies.

The study conducted by Mousa et al. [27] aimed at detecting IL-13 expression in BC tumor tissues and explaining the relationship between its expression and the clinical form of cancer. Expression of IL-13 in tumor cells has been studied in patients with bladder cancer at various stages of clinical malignancy and patients with benign bladder tumors. Tissue sections were analyzed by immunohistochemistry for the presence of IL-13. The results showed a high positive expression of IL-13 in high-grade cancer tissues compared to tissues from benign tumors, which confirmed that IL-13 is involved in tumor progression. High levels of IL-13 provoke reduced tumor immune control, which leads to unrestrained tumor growth. Involved in immune surveillance, the IL-13 tumor contributes to escape from apoptosis and strengthens its growth [28].

To summarize, the obtained results indicate the important role of the examined parameters of angiogenesis, oxidative stress, and inflammation in the pathogenesis and development of BC. Additionally, differences in terms of structure and function revealed by the mutual correlations between examined parameters in the urine in BC patients may indicate their complicity in the tumor process occurring within urinary bladder cancer.

A major aim in the management of urothelial carcinoma is the prevention of the recurrence and progression of the disease. For patients with an intermediate-to-high risk of recurrence, the standard treatment consists of intravesical instillations with bacillus Calmette–Guérin (BCG) to prevent or delay tumor recurrence and progression [29].

Ferro et al. [30] studied the association of baseline counts of basophils, eosinophils, and monocytes with outcomes of patients with high-grade T1 bladder cancer receiving a standard course of intravesical BCG. Baseline basophil count may predict recurrence in BCG-treated HG/G3 T1 bladder cancer patients. Basophils may have a role in BCG-treated bladder cancer patients because of their ability to facilitate Th2 polarization by secreting proinflammatory interleukins (IL-4 or IL-13) and skewing antigen-presenting cells towards a type 2 response.

Angiogenesis is a crucial step for tumor growth and progression in almost all types of cancers, including BC [31]. In cancer tissues, microvessel density (MVD) is commonly used to evaluate the angiogenic status. MVD was found to be closely associated with recurrence after intravesical BCG therapy [32].

BCG serves as a free radical generator. BCG generates H_2_O_2_ which serves as the trigger for COS and cell damage. Production of H_2_O_2_ by BCG, following binding to and internalization by BC cells, sets the stage for a second wave of cell-generated oxidants involving iNOS [33]. Free radicals (superoxide and NO) and reactive molecules potentiate intracellular signaling pathways and downstream gene expression and result in a cellular phenotype that defines the BCG treatment effect. Loss of BCG viability is associated with decreased H_2_O_2_ production, inefficient induction of COS and its direct consequences, and decreased treatment efficacy [34]. NO is considered one of the main factors responsible for the cytotoxic activity that macrophages exert against tumor cells [31].

Based on the data presented above, the use of the urinary parameter panel (characterizing processes such as angiogenesis, immune response, and oxidative stress) seems to be useful in assessing the effectiveness of BCG therapy in patients with BC. The increase in IL-13, ANG, and SNCG in the urine of BC patients may be an unfavorable prognostic factor in BCG therapy, in contrast to ANGST and 8-iso-PGF2*α*, proving the treatment effectiveness.

Obesity and metabolic disorder such as type 2 diabetes mellitus (T2DM) have been identified as a major risk factor associated with cancers, including BC. The incidence of cancer due to obesity is estimated to be approximately 20% of all causes of cancers [35].

Ferro et al. [36] investigated the prognostic role of T2DM in patients with primary T1HG/G3 nonmuscle-invasive bladder cancer (NMIBC) treated with transurethral resection of the bladder (TURB) and BCG therapy. The authors showed that prior history of T2DM was significantly associated with a greater risk of disease recurrence and disease progression to muscle-invasive BC, as well as with overall and cancer-specific survival.

Meta-analysis of 14 prospective cohort studies showed a nonlinear positive relationship between the BMI and bladder cancer suggesting that each 5 kg/m^2^ increase of the BMI corresponded to a 3.1% increase of the bladder cancer risk, especially when the BMI exceeded 30 kg/m^2^ [37].

Ferro et al. [38] evaluated the impact of the BMI on survival in patients with high-risk nonmuscle-invasive bladder cancer. In particular, they showed that overweight and obesity were associated with a greater risk of progression. Instead, a higher risk of recurrence was demonstrated only for obese patients. The association of obesity with a poor clinical outcome in T1G3 NMIBC could be explained on the basis of several factors. In particular, it is well known that obesity is characterized by insulin resistance and low-grade systemic inflammation, which may affect the oncological outcomes of NMIBC patients as a result of insulin, insulin-like growth factor 1(IGF-1), cytokines, and growth factor effects. Furthermore, obesity is associated with high levels of inflammatory cytokines, such as leptin, IL-6, and TNF-*α*, produced by adipocytes and immune cells infiltrating adipose tissue.

To date, there is no reliable method for the prediction of response to chemotherapy, resulting in a possible overtreatment in nonresponders with unnecessary toxicity that might render patients in a deteriorated physical condition without the opportunity for additional, alternative therapy [39]. The parameters presented in our study, determined in the urine of patients with BC, reflect the multifaceted processes occurring during the course of the disease, such as angiogenesis, oxidative stress, and immune response. So far, a similar parameter panel has not been investigated in a possible evaluation of the effectiveness of chemotherapy and immunotherapy in bladder cancer. Considering the noninvasive nature of the tests (urine tests), which are an important alternative to invasive tests, it seems interesting to investigate the changes in the concentration of selected parameters before and during the subsequent stages of the therapy. Such studies could assess the multidirectional influence of chemotherapy and immunotherapy in BC.

## 5. Conclusion

The levels of selected urinary parameters (ANG, ANGST, 8-iso-PGF2*α*, SNCG, and IL-13) were statistically significantly higher than the levels of these parameters observed in the control group. The presented results may suggest the association of oxidative stress (reflected by lipid peroxidation), angiogenesis process, or inflammation in bladder cancer pathogenesis and the development of this disease. It is reasonable to continue research in order to thoroughly assess the impact of various associated processes on the course of this disease.

The possibility of using a panel of selected urinary BC parameters to assess the effectiveness of BCG therapy should also be considered.

## Figures and Tables

**Table 1:** Demographic and clinical data of the bladder cancer (BC) patient group and control group.

Population characteristic	BC group	Controls
*N*	60	28
Age range (median)	41–88 (66)	50–81 (67)
Male	50 (83%)	23 (82%)
Female	10 (17%)	5 (18%)
*Clinical grading*		
LG	31 (52%)	
HG	29 (48%)	
*Clinical subgroups*		
NMIBC	50 (83%)	
MIBC	10 (17%)	
Primary cancer	29 (48%)	
Cancer recurrence	31 (52%)	

*N*: number of patients; LG: low grade; HG: high grade; NMIBC: nonmuscle-invasive bladder cancer; MIBC: muscle-invasive bladder cancer.

**Table 2:** Median levels and interquartile ranges of examined parameters in the urine of the BC group and control group with statistical analysis.

Parameters	BC group		Control group		*p* value
	Median	IQR	Median	IQR	
ANG (ng/mg cr.)	0.58	0.31–1.04	0.06	0.04–0.15	<0.001*∗*
ANGST (ng/mg cr.)	0.53	0.28–0.91	0.08	0.06–0.16	<0.001*∗*
8-Iso-PGF2*α* (ng/mg cr.)	5.11	2.90–25.58	3.44	2.74–4.58	0.013*∗*
SNCG (pg/mg cr.)	15.84	13.05–26.88	12.41	9.06–16.07	0.001*∗*
IL-13 (pg/mg cr.)	1.81	1.23–3.21	1.01	0.68–1.58	<0.001*∗*

ANG: angiogenin; ANGST: angiostatin; 8-Iso-PGF2*α*: 8-iso-prostaglandin F2*α*; SNCG^:^*ɣ*-synuclein; IL-13: interleukin-13; IQR: interquartile ranges. *∗*Statistically significant difference between BC and the control group (*U* Mann–Whitney test).

**Table 3:** Diagnostic value of examined parameters.

Diagnostic value of parameters	Se (%) (CI 95%)	Sp (%) (CI 95%)	PPV (%) (CI 95%)	NPV (%) (CI 95%)	LR+ (CI 95%)	LR− (CI 95%)
ANG (ng/mg cr.)	83.3 (71.5–91.0)	92.9 (76.5–99.1)	96.2 (86.7–99.0)	72.2 (59.4–82.2)	11.7 (3.1–44.6)	0.2 (0.1–0.3)
ANGST (ng/mg cr.)	71.7 (58.6–82.5)	96.4 (81.7–99.9)	97.7 (86.2–99.7)	61.4 (51–70.5)	20.1 (2.9–138.4)	0.2 (0.2–0.4)
8-Iso-PGF2*α* (ng/mg cr.)	45.0 (32.1–58.4)	96.4 (81.7–99.9)	96.4 (79.4–99.5)	45.0 (39.2–51.0)	12.6 (1.8–88.1)	0.6 (0.4–0.7)
SNCG (pg/mg cr.)	71.7 (58–82.5)	71.4 (51.3–86.8)	84.3 (74.6–90.8)	54.1 (42.5–65.2)	2.5 (1.4–4.6)	0.4 (0.2–0.6)
IL-13 (pg/mg cr.)	80.1 (67.7–89.2)	60.7 (40.6–78.5)	81.4 (73.0–87.6)	58.6 (44.1–71.8)	2.0 (1.3–3.3)	0.3 (0.2–0.6)

ANG: angiogenin; ANGST: angiostatin; 8-Iso-PGF2*α*: 8-iso-prostaglandin F2*α*; SNCG: *ɣ*-synuclein; IL-13: interleukin-13; Se: sensitivity; Sp: specificity; PPV: positive predictive value; NPV: negative predictive value; LR+: positive likelihood ratio; LR−: negative likelihood ratio; CI: confidence interval.

**Table 4:** Median levels and interquartile ranges of parameters in the urine of the LG (A) subgroup, HG (B) subgroup, and control group (C) with statistical analysis.

Parameters	Subgroups							
	LG (A)		HG (B)		Control group (C)		*p* value	Post hoc analysis
	Me	IQR	Me	IQR	Me	IQR		
ANG (ng/mg cr.)	0.42	0.25–0.81	0.72	0.33–1.31	0.06	0.04–0.15	<0.001	A : C < 0.001*∗* B : C < 0.001*∗* A : B = NS
ANGST (ng/mg cr.)	0.41	0.24–0.71	0.54	0.45–1.13	0.08	0.06–0.16	<0.001	A : C < 0.001*∗* B : C < 0.001*∗* A : B = NS
8-Iso-PGF2*α* (ng/mg cr.)	5.15	3.10–33.48	5.07	2.72–17.60	3.44	2.74–4.58	0.03	A : C = 0.03*∗* B : C = NS A : B = NS
SNCG (pg/mg cr.)	17.11	12.67–28.61	15.32	13.97–25.97	12.41	9.06–16.07	0.002	A : C = 0.004*∗* B : C = 0.013*∗* A : B = NS
IL-13 (pg/mg cr.)	1.58	1.11–3.57	1.88	1.24–2.75	1.01	0.68–1.58	<0.001	A : B = 0.004*∗* B : C = 0.002*∗* A : B = NS

ANG: angiogenin; ANGST: angiostatin; 8-Iso-PGF2*α*: 8-iso-prostaglandin F2*α*; SNCG: *ɣ*-synuclein; IL-13: interleukin-13; LG: low grade; HG: high grade; Me: median; CI: confidence interval; NS: not statistically significant. *∗*Statistically significant difference between LG (A), HG (B), and control group (C) using Kruskal–Wallis test.

**Table 5:** Median levels and interquartile ranges of examined parameters in the urine of the NMIBC (A) subgroup, MIBC (B) subgroup, and control group with statistical analysis.

Parameters	Subgroups							
	NMIBC (A)		MIBC (B)		Control group (C)		*p* value	Post hoc analysis
	Me	IQR	Me	IQR	Me	IQR		
ANG (ng/mg cr.)	0.46	0.31–0.97	0.80	0.59–1.07	0.06	0.04–0.15	<0.001	A : C < 0.001*∗* B : C < 0.001*∗* A : B = NS
ANGST (ng/mg cr.)	0.50	0.27–0.80	0.78	0.49–1.53	0.08	0.06–0.16	<0.001	A : C < 0.001*∗* B : C < 0.001*∗* A : B = NS
8-Iso-PGF2*α* (ng/mg cr.)	5.11	3.10–31.36	4.27	2.72–18.34	3.44	2.74–4.58	0.04	A : C = 0.03*∗* B : C = NS A : B = NS
SNCG (pg/mg cr.)	15.83	12.67–28.61	16.00	14.50–25.97	12.41	9.06–16.07	0.002	A : C = 0.004*∗* B : C = 0.024*∗* A : B = NS
IL-13 (pg/mg cr.)	1.67	1.20–3.04	2.23	1.32–3.84	1.01	0.68–1.58	<0.001	A : B = 0.002*∗* B : C = 0.004*∗* A : B = NS

ANG: angiogenin; ANGST: angiostatin; 8-Iso-PGF2*α*: 8-iso-prostaglandin F2*α*; SNCG: *ɣ*-synuclein; IL-13: interleukin-13; NMIBC: nonmuscle-invasive bladder cancer; MIBC: muscle-invasive bladder cancer; Me: median; CI: confidence interval; NS: not statistically significant. *∗*Statistically significant difference between NMIBC (A), MIBC (B), and control group (C) using Kruskal–Wallis test.

**Table 6:** Median levels and interquartile ranges of examined parameters in the urine of the BC primary cancer (A) subgroup, the cancer recurrent (B) subgroup, and the control group (C) with statistical analysis.

Parameters	Subgroups							
	Primary cancer (A)		Cancer recurrent (B)		Control group (C)		*p* value	Post hoc analysis
	Me	IQR	Me	IQR	Me	IQR		
ANG (ng/mg cr.)	0.57	0.32–1.31	0.68	0.30–0.92	0.06	0.04–0.15	<0.001	A : C < 0.001*∗* B : C < 0.001*∗* A : B = NS
ANGST (ng/mg cr.)	0.56	0.33–1.14	0.49	0.27–0.75	0.08	0.06–0.16	<0.001	A : C < 0.001*∗* B : C < 0.001*∗* A : B = NS
8-Iso-PGF2*α* (ng/mg cr.)	4.79	3.31–32.11	5.89	2.26–19.97	3.44	2.74–4.58	0.04	A : B = NS B : C = NS A : B = NS
SNCG (pg/mg cr.)	16.68	13.09–27.67	15.46	13.02–23.31	12.41	9.06–16.07	0.002	A : B = 0.006*∗* B : C = 0.009*∗* A : B = NS
IL-13 (pg/mg cr.)	1.81	1.25–3.37	1.79	1.21–2.85	1.01	0.68–1.58	<0.001	A : B = 0.004*∗* B : C = 0.002*∗* A : B = NS

ANG: angiogenin; ANGST: angiostatin; 8-Iso-PGF2*α*: 8-iso-prostaglandin F2*α*; SNCG: *ɣ*-synuclein; IL-13: interleukin-13; NMIBC: nonmuscle-invasive bladder cancer; MIBC: muscle-invasive bladder cancer; Me: median; CI: confidence interval; NS: not statistically significant. *∗*Statistically significant difference between primary cancer (A), cancer recurrence (B), and control group (C) using Kruskal–Wallis test.

## Data Availability

The datasets generated and/or analyzed during the current study are available from the corresponding author upon reasonable request.
